# Efficacy of 0.05% cyclosporine A on the lipid layer and meibomian glands after cataract surgery: A randomized, double-masked study

**DOI:** 10.1371/journal.pone.0245329

**Published:** 2021-01-11

**Authors:** Min Seung Kang, Jonghoon Shin, Jeong Min Kwon, Jin Huh, Ji Eun Lee

**Affiliations:** 1 Department of Ophthalmology, Pusan National University School of Medicine, Yangsan, South Korea; 2 Department of Ophthalmology, Research Institute for Convergence of Biomedical Science and Technology, Pusan National University Yangsan Hospital, Yangsan, South Korea; 3 Department of Ophthalmology, Busan Bohun Veterans, Busan, Korea; 4 Department of Convergence Medicine, Pusan National University School of Medicine, Yangsan, South Korea; University of California Berkeley, UNITED STATES

## Abstract

**Purpose:**

To quantitatively evaluate the effects of 0.05% cyclosporine A (CsA) on lipid layer thickness (LLT) and meibomian glands after cataract surgery using the LipiView^®^ ocular surface interferometer.

**Methods:**

This study was a prospective randomized double-masked clinical trial conducted by Pusan National University Yangsan Hospital between April 04, 2019, and November 31, 2019. Sixty-two participants were recruited, and 12 of them were not enrolled because they had undergone previous treatments for ocular surface diseases. The participants were adult patients with cataract, exhibiting normal lid position; they did not present any other ocular disease and did not meet the exclusion criteria of the clinical trial. Fifty subjects were enrolled in the study. The randomized subjects received treatment with 0.05% CsA (group A) or 0.5% carboxymethyl cellulose (CMC) (group B) over the 3 months following the cataract surgery. Subjective and objective assessments were performed at preoperative and postoperative visits. Ocular Surface Disease Index (OSDI), tear breakup time (TBUT), and Schirmer’s I test were performed by the same surgeon, and LLT and meiboscore were determined using the LipiView^®^ interferometer.

**Results:**

Fifty subjects subjects enrolled consisted of men (50%) and women (50%), with a mean (SD) age of 65.94 (10.35) years. Four subjects in group A and five in group B were excluded from the analysis as they were lost to follow-up within 1 month after cataract surgery. Thus, the study comprised 41 eyes of 41 subjects; 21 subjects were treated with CsA and 20 subjects with CMC. Comparing the clinical measurements between groups A and B taken at the last visit, while controlling the effects of the preoperative values, TBUT and LLT showed significant differences (*p* = 0.035 and *p* = 0.047, respectively, by ANCOVA). The TBUT between the subjects using CsA and those using CMC after cataract surgery showed a significant difference during follow up (*p* = 0.003 by repeated measures ANOVA). In the multivariate analysis, preoperative LLT and the use of CsA were found to be independent parameters for postoperative LLT (R^2^ = 0.303; *p* = 0.008 and *p* = 0.045, respectively), whereas the follow-up duration exhibited a positive correlation with the difference between the preoperative and postoperative values of LLT in the group treated with CsA (R^2^ = 0.738 and *p* < 0.001).

**Conclusion:**

Treatment with 0.05% CsA following cataract surgery is effective in improving TBUT and LLT in comparison with 0.5% CMC. A higher preoperative value of LLT and the postoperative use of CsA could be significant determinants of a higher postoperative LLT value.

**Trial registration:**

ISRCTN registry with ISRCTN 10173448.

## Introduction

Cataract is the principal cause of decreased visual acuity in the geriatric population, and surgical treatment is widely preferred owing to its effectiveness and safety [1, 2]. However, apart from improving visual acuity, surgical treatment often leads to discomfort associated with iatrogenic dry eye syndrome [3–5]. Numerous studies have reported that the incidence of dry eye syndrome after cataract surgery is 9.8% to 42% postoperatively [4, 6]. Complications after cataract surgery such as dry eye syndrome can occur because of multiple factors, including corneal nerve transection, inflammation, goblet cell loss, and meibomian gland dysfunction (MGD) [3, 7]. In particular, the occurrence of MGD could be one of the main causes of dry eye syndrome after cataract surgery [7, 8].

MGD is caused by the blockage of the meibomian gland (MG) orifice due to the solidification of secretions or hyperkeratinization of the epithelium [9]. This causes a decrease in tear lipid layer thickness (LLT), potentially resulting in evaporative dry eye. Various therapies have been suggested to manage the symptoms of MGD, such as warm compresses and squeezing and wiping the lid; however, a complete remission of symptoms is rare [10, 11]. Cyclosporine A (CsA), a calcineurin inhibitor, has been recently introduced as the drug of choice for MGD owing to its anti-inflammatory effect caused by the suppression of T cell-mediated immune responses [12]. Many studies have reported that CsA alone or in combination with other therapies is effective in improving MG function [13–15]. To our knowledge, no report exists on the effect of 0.05% CsA on the tear lipid layer and MG following cataract surgery. Additionally, an objective evaluation of the tear lipid layer and MG grading using an image-based diagnostic tool (such as LipiView^®^) have not yet been conducted to measure the efficacy of 0.05% CsA. Therefore, we aimed to quantitatively evaluate the efficacy of 0.05% CsA on LLT and MG after cataract surgery using the LipiView^®^ interferometer.

## Materials and methods

The protocol for this clinical trial and the supporting CONSORT checklist are available as supporting information files (S1 Checklist, S1 and S2 Files, and S1 Table). This was a prospective, randomized, double-masked, controlled clinical trial evaluating the efficacy of 0.05% CsA on the lipid layer and MG in subjects after cataract surgery. The study protocol was approved by the Pusan National University Yangsan Hospital Institutional Review Board, and the study was performed in accordance with the tenets of the Declaration of Helsinki. The subjects provided informed consent to participate in the study. The recruitment start date for the study was April 04, 2019, and the recruitment end date was May 31, 2019. The study was conducted between April 04, 2019, and November 31, 2019, at Pusan National University Yansan Hospital, Yangsan, Korea, and was registered with the International Standard Randomized Controlled Trial Number (ISRCTN 10173448).

### Subjects

The subjects included in the study were adults with cataract who exhibited normal lid position and closure and did not have any ocular diseases. We excluded subjects who had used topical artificial tears, anti-inflammatory agents, antibiotics, or other medications that could dry out the eye or stimulate tear secretion during the 90-day period before the surgery. Eyes with a history of trauma, ocular surgery, laser or systemic treatment known to affect tear secretion, autoimmune disease, current use of contact lenses, and/or history of slit-lamp evidence of eye surface disorders were also excluded from the study. In addition, to exclude subjects with dry eyes from the study, the subjects were required to show a normal fluorescent tear breakup time (TBUT) (> 10 seconds) and Schirmer’s I test result (> 10 mm with anesthesia) preoperatively.

In total, 62 participants (62 eyes) were recruited, and only 50 subjects (50 eyes) were enrolled for the study at the Department of Ophthalmology of Pusan National University Yangsan Hospital. Twelve initial recruits were not included because they had undergone previous treatments for ocular surface diseases. The sample size was calculated using MedCalc version 10.0 (MedCalc Software; Ostend, Mariakerke, Belgium). The minimum required sample size for a *t*-test with an alpha level of 0.05 and a power of 0.8 was calculated to be 21 for each group; considering a 20% dropout rate, 25 participants were enrolled in each group. Four subjects in group A and five subjects in group B were excluded from the analysis as they were lost to follow up within 1-month after cataract surgery.

Eligible subjects were enrolled in the study and assigned a sequential number with a corresponding randomization code generated by an independent third party using the SAS software (version 8.0, SAS Institute Inc., Cary, NC, USA). According to the randomization, the clinical staff assigned the subjects to receive either 0.05% CsA ophthalmic emulsion (Restasis^®^, Allergan Inc., Irvine, CA, USA) or 0.5% carboxymethyl cellulose (CMC) (Refresh plus®, Allergan Inc.) twice daily for 3 months after cataract surgery. The clinical staff provided instructions on how to administer the ophthalmic solutions. To achieve masking of the researchers and subjects, the medications were filled by a pharmacologist, and the type of each topical medication was not revealed until after completing the follow-up examination at the end of the study.

All subjects underwent standard small-incision cataract surgery performed by the same surgeon (JEL). A clear corneal incision 2.8 mm in length was made at the temporal region in the eye. All eyes received identical postoperative eyedrops with a combination of levofloxacin 1.5% four times daily for 2 weeks, fluorometholone 0.1% four times daily for 1 week, followed by weekly tapering doses and either CsA or CMC twice daily for 3 months.

### Clinical measurements

To assess ocular surface status, the following measurements were performed during the follow-up period in all subjects. TBUT and Schirmer’s I test score were determined both preoperatively and postoperatively after 1 month, 3 months, and at the last visit; the Ocular Surface Disease Index (OSDI) questionnaire, used to evaluate the subjects’ symptoms, and LLT using an interferometer were evaluated preoperatively and postoperatively at the last visit. Minimum, maximum, and average tear LLT were measured using the LipiView® Ocular Surface Interferometer (TearScience® Inc, Morrisville, NC, USA). Subjects with interferometer results quality < 0.8 were excluded from the study. The degree of MG dropout was calculated as meiboscores for the upper and lower eyelids using the LipiView^®^ interferometer, according to the grading scale described by Arita et al. [16]: grade 0, no atrophy; grade 1, 1% to 33% atrophy; grade 2, 34% to 66% atrophy; and grade 3, more than 66% atrophy.

The primary outcomes were the changes in TBUT, Schirmer’s I test score, OSDI questionnaire score, and LLT between the preoperative and postoperative last visit in the 0.05% CsA treatment group and CMC control group. The secondary outcomes were the changes in TBUT and Schirmer’s I test score during the follow-up period between the treatment and control groups and the baseline factors that affect LLT in each group. The TBUT measurement was conducted and the OSDI questionnaire was administered by a single observer (JEL).

### Statistical analysis

All statistical analyses were performed using SPSS for Windows 26.0 (SPSS Inc, Chicago, IL, USA). Descriptive statistics were presented as the mean standard deviation (SD). Data normality was verified using the Kolmogorov-Smirnov method before performing the analysis of covariance (ANCOVA) and repeated measures analysis of variance (ANOVA). The ANCOVA, including normality of the dependent variable, homogeneity of variance, and the linear relationship between the covariate and dependent variable, was checked and confirmed; therefore, we used the ANCOVA for comparing the mean value of the postoperative variables between the two groups while statistically controlling the effects of the preoperative variables.

The time course of statistical changes in the value of TBUT and Schirmer’s I test score between the two groups was evaluated by repeated measures ANOVA. Multiple linear regression analysis was used to identify the determinant factors associated with the LLT measurements. Each variable was initially analyzed using a univariate model; all significant variables were subsequently evaluated using a multivariate model by the backward method. The relationships between LLT parameters and associated factors were additionally examined using scatter plots and linear regression. The coefficient of determination (R^2^) in the linear regression was reported, and *p* values < 0.05 were considered statistically significant.

## Results

**Fig 1:**
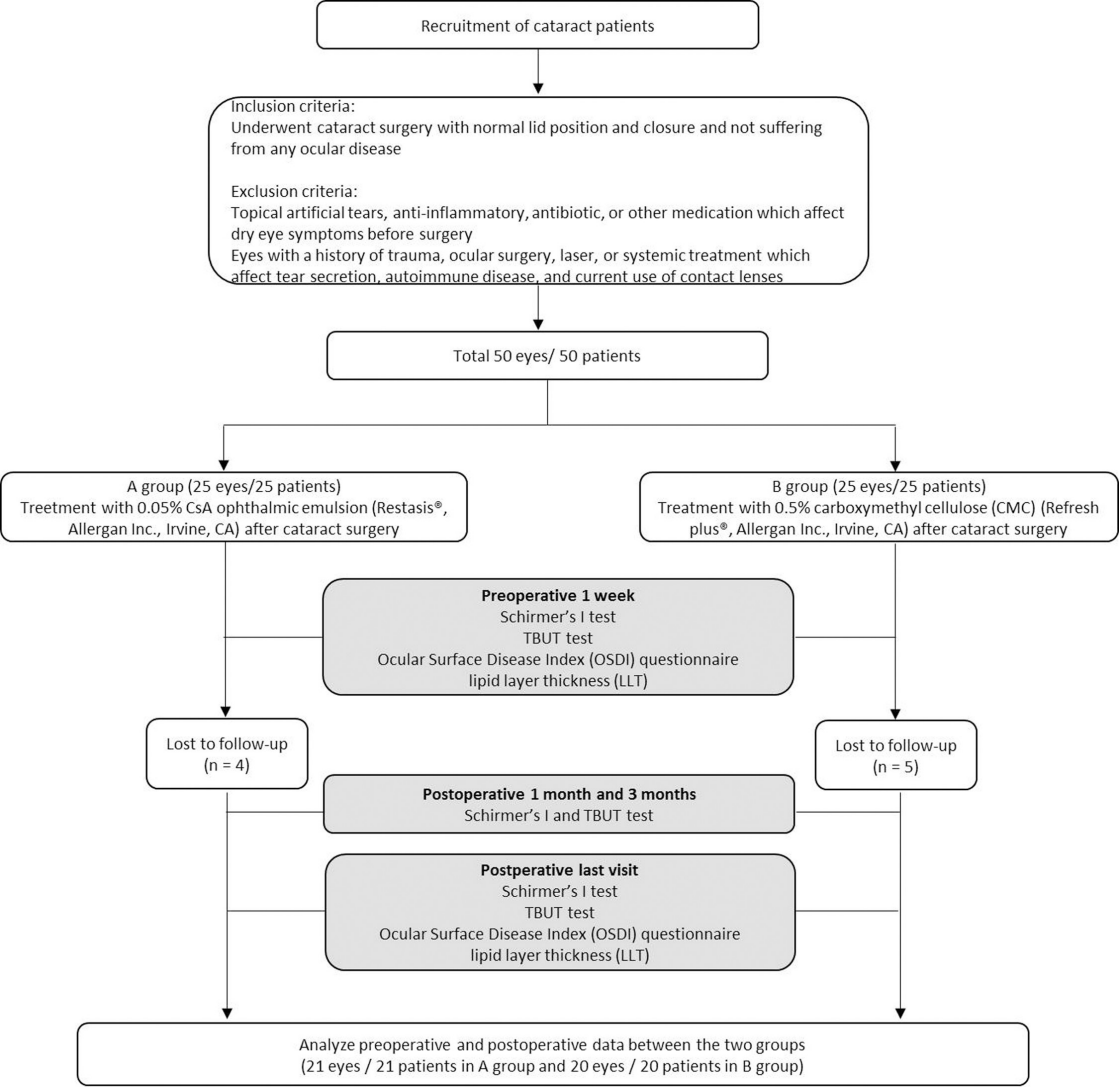
CONSORT flowchart of the study.

Fifty subjects (50 eyes) were enrolled in the study. Four subjects in group A and five subjects in group B were excluded from each group as they were lost to follow up within 1 month after cataract surgery. Finally, 21 subjects treated using CsA in group A and 20 subjects treated using CMC in group B were analyzed (Fig 1). No subject exhibited complications or side effects at any visit during the study, including allergy or intolerance to any of the products used in the study.

Table 1 shows the clinical characteristics and demographics at the preoperative visit in groups A and B. There were no statistically significant differences between the groups in terms of demographic data.

**Table 1:** Preoperative clinical characteristics and demographics.

	Group A		Group B		
	Mean (SD)	95%, CI	Mean (SD)	95%, CI	*p* value
Age (yr)	63.43 (12.09)	57.92, 68.93	68.45 (8.83)	64.31, 72.58	0.136*
TBUT (sec)	10.76 (1.13)	10.24, 11.28	11.05 (1.23)	10.47, 11.62	0.307*
Schirmer’s I test score (mm)	14.62 (5.66)	12.04, 17.20	15.80 (5.0)	13.45, 18.14	0.127*
OSDI score	18.08 (6.42)	14.00, 22.16	13.06 (9.41)	8.37, 17.34	0.093*
LLT (nm)	77.67 (21.71)	67.78, 87.54	75.80 (20.56)	66.17, 85.42	0.660*
Follow-up duration (days)	132.24 (27.65)	119.65, 144.82	128.10 (18.10)	119.63, 136.57	0.576*
	N (%)		N (%)		
Gender (male/female)	10 (47.6) / 11 (52.4)		13 (65.0) / 7 (35.0)		0.262^#^
Laterality (OD/OS)	13 (61.9) / 8 (38.1)		7 (35.0) / 13 (65.0)		0.085^#^
Upper meiboscore (grade 0/1/2/3)	6 (28.6) / 6 (28.6) / 6 (28.6) / 3 (14.3)		6 (30.0) / 5 (25.0) / 8 (40.0) / 1 (5.0)		0.717^#^
Lower meiboscore (grade 0/1/2/3)	6 (28.6) / 6 (28.6) / 6 (28.6) / 3 (14.3)		4 (20.0) / 8 (40.0) / 6 (30.0) / 2 (10.0)		0.835^#^

TBUT: tear breakup time.

OSDI score: ocular surface disease index score.

LLT: lipid layer thickness.

* Results of continuous parameters between the two groups using an independent *t*-test.

^#^ Results of non-continuous parameters between the two groups using chi-square analysis.

Table 2 presents the values of TBUT, Schirmer’s I test score, OSDI score, LLT, and meiboscore at the preoperative and last postoperative visits in both groups. Comparing the clinical measurements at the last visit between groups A and B, TBUT and LLT, while controlling the effects of the preoperative values, showed significant differences between the two groups (*p* = 0.035 and *p* = 0.047, respectively by ANCOVA).

**Table 2:** Comparison of clinical measurements between the preoperative and postoperative last visits.

	Group A		Group B		
	Preoperative	Last	Preoperative	Last	
	Mean (SD)	Mean (SD)	Mean (SD)	Mean (SD)	*p* value*
TBUT (sec)	10.76 (1.13)	11.95 (1.59)	11.05 (1.23)	10.90 (1.71)	0.034*
Schirmer’s I test score (mm)	14.62 (5.66)	15.33 (3.99)	15.80 (5.0)	15.70 (5.36)	0.861
OSDI score	18.08 (6.42)	7.60 (7.96)	13.06 (9.41)	7.00 (4.37)	0.736
LLT (nm)	77.67 (21.71)	81.57 (18.45)	75.80 (20.56)	71.45 (19.64)	0.047*

TBUT: tear breakup time.

OSDI score: ocular surface disease index score.

LLT: lipid layer thickness.

* Results of the between-group analysis using an ANCOVA (with the preoperative measurements as the covariate).

**Fig 2:**
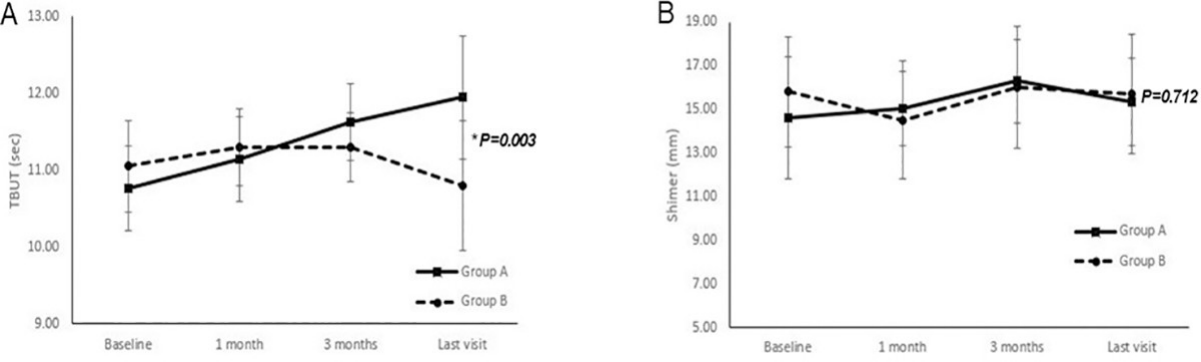
Changes in tear breakup time (TBUT) (A) and Schirmer’s I test score (B) during the follow up in each group. The TBUT at the follow up after cataract surgery was significantly different between the subjects using 0.05% cyclosporine A and carboxymethylcellulose (*p* = 0.003 by repeated measures ANOVA). The last visit ranged from 96 days to 180 days in group A and 99 days to 160 days in group B.

The TBUT measurement during the follow up after cataract surgery was significantly different between the two groups, whereas Schirmer’s I test score was not (*p* = 0.003 and *p* = 0.712, respectively, by repeated measures ANOVA) (Fig 2).

**Fig 3:**
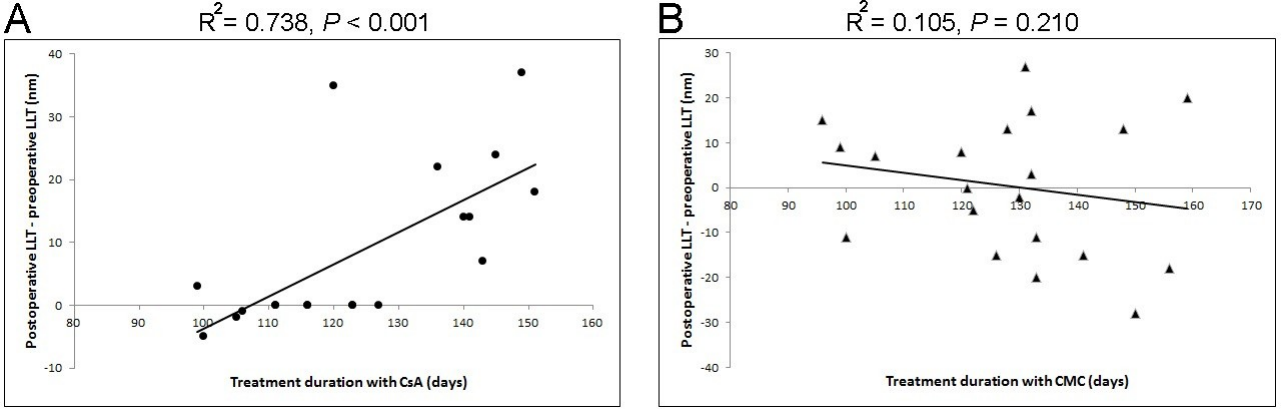
Correlation between preoperative and postoperative Lipid Layer Thickness (LLT) and treatment duration in each group. The scatterplot displays the influence of the duration of treatment with 0.05% cyclosporine A (CsA) (A) and carboxymethylcellulose (CMC) (B) on the difference between the preoperative and postoperative LLT. The difference between the preoperative and postoperative LLT is significantly correlated with the duration of 0.05% CsA use; however, it is not significantly correlated with the duration of CMC use (*p* < 0.001 and *p* = 0.210, respectively).

Multivariate linear regression analysis was performed to examine the influence of independent preoperative parameters on the postoperative LLT. Preoperative LLT and the subjects using CsA were independent parameters for postoperative LLT (R^2^ = 0.303; *p* = 0.008 and *p* = 0.045, respectively) (Table 3). In addition, the follow-up duration showed a significantly positive correlation with the difference between the preoperative and postoperative values of LLT in only group A (R^2^ = 0.738 and *p* < 0.001); however, the correlation was not significant in group B (R^2^ = 0.105 and *p* = 0.21) (Fig 3).

**Table 3:** Univariate and multivariate linear regression analysis to examine the influence of the independent preoperative parameters on postoperative Lipid Layer Thickness (LLT).

	Univariate		Multivariate	
	ß (95% CI)	*p* value	ß (95% CI)	*p* value*
Age (yr)	0.164 (-0.417, 0.746)	0.571		
Group (reference: Group B)	**14.407 (3.009, 25.805)**	**0.015**	**13.244 (0.314, 26.174)**	**0.045**
Preoperative TBUT (sec)	-1.145 (-6.418, 4.128)	0.663		
Preoperative Schirmer’s I (mm)	-0.304 (-1.472, 0.863)	0.601		
Preoperative OSDI score	0.031 (-0.848, 0.910)	0.943		
Preoperative LLT (nm)	**0.443 (0.170, 0.696)**	**0.002**	**0.167 (0.116, 0.720)**	**0.008**
Preoperative lower meiboscore	- 1.272 (-7.612, 5.069)	0.697		
Follow-up duration (days)	0.067 (-0.201, 0.333)	0.616		

TBUT: tear breakup time.

OSDI score: ocular surface disease index score.

Statistically significant values are in bold type font.

* Result of the backward method in the multivariate analysis.

## Discussion

In the present study, postoperative treatment with 0.05% CsA following cataract surgery improved TBUT and LLT compared with the controls. In addition, higher preoperative LLT and 0.05% CsA treatment after cataract surgery were significantly associated with increased LLT at the postoperative follow-up visit.

Hamada et al. reported that subjects treated with 0.05% CsA following cataract surgery showed greater improvement than subjects treated without CsA with regard to tear osmolarity, ocular surface staining, TBUT, and the results of Schirmer’s I tests [17]. In addition, Chung et al. suggested that 0.05% CsA would be an effective and safe treatment for dry eye after cataract surgery [18]. LipiView^®^ is a recently developed device that can quantitatively measure tear film LLT using interferometry, reported to be a beneficial tool for evaluating the ocular surface [19, 20]: it can objectively measure the efficacy of 0.05% CsA treatment following cataract surgery. To our knowledge, this is the first study to evaluate the efficacy of postoperative 0.05% CsA therapy after cataract surgery by quantitatively assessing LLT and meiboscore as well as TBUT, Schirmer’s I test score, and the OSDI questionnaire score.

The clinical parameters of TBUT and LLT showed significant differences between the two groups at the last visit. It was reported that in subjects treated with 0.05% CsA after cataract surgery, TBUT showed a significant improvement compared with the control group [17, 18]. Cataract surgery could serve as a risk factor for postoperative dry eye disease that could occur as a result of corneal nerve transection, inflammation, goblet cell loss, and MGD [3, 7, 21, 22]. As 0.05% CsA may decrease MG inflammation, the symptoms of MGD and MG plugging may decrease owing to the highly specific immunomodulating process affecting T lymphocytes; 0.05% CsA can effectively control ocular surface inflammation and treat MGD [23–25]. Decreased LLT has been associated with the instability of the lipid layer, and MGD could contribute to the emergence of ocular surface disease (OSD) [5, 26]; therefore, in this study, the significant difference in postoperative LLT between eyes treated with and without CsA could be attributed to the improvement in the ocular surface caused by postoperative CsA.

The Schirmer’s I test results did not show significant differences between the two groups. This test is prone to variable results, poor reproducibility, and low sensitivity for detecting dry eye; therefore, the result of this test did not appear to accurately reflect changes in the tear film [27]. In addition, the OSDI scores did not show significant differences between preoperative and postoperative visits in both groups. Among the questions in the OSDI questionnaire, two items related to blurred and poor vision can induce bias due to better visual acuity postoperatively; therefore, the OSDI score may have limitations in comparing the tear film between two groups after cataract surgery [4]. Since we excluded underlying dry eye syndrome preoperatively through objective measurements (TBUT and Schirmer’s I test score) without the OSDI score, it might influence the result. However, we compared preoperative and postoperative measurements; thus, the results might indicate whether subjective symptoms can be improved by using the eye drops.

The present study showed that thinner preoperative LLT could be a significant determinant of an even thinner postoperative LLT. These results indicate that subjects with preoperative severe ocular surface disturbances exhibit a higher risk of developing postoperative complications, and these complications could be associated with poor visual quality and postoperative subject dissatisfaction [3, 28]. In their review, Movahedan and Djalilian noted that the preoperative management of OSD plays an essential role in reducing postoperative complications [29]. Several previous studies have revealed that cataract surgery can lead to dry eye, and such ocular discomfort is often associated with reduced postoperative visual acuity. Hence, as postoperative management with 0.05% CsA following cataract surgery can improve ocular surface disturbances and the symptoms of OSD, it could also lead to increased subject satisfaction [3, 28]. The present study found that a longer duration of treatment with 0.05% CsA is significantly associated with higher values of LLT after cataract surgery; therefore, continuing treatment with CsA could improve dry eye-related discomfort following cataract surgery.

Although this study has the advantages of a double-masked, randomized, prospective assessment, which was designed to minimize bias, it also has some limitations. First, this study was conducted on a small number of subjects in each group and with a relatively short-term follow up. Second, 0.5% CMC was used as the control instead of a vehicle for 0.05% CsA because the latter was not available. Because there is no comparison between a vehicle for 0.05% CsA and CMC, the emulsion effect of the CsA vehicle in group A might have influenced the study results. Third, the meiboscore evaluates only morphological changes in the MG, and it cannot detect quality changes in the meibum. The relative significance of these quality changes in the meibum and the morphologic changes in the MGs remains to be clarified. Further studies are necessary to confirm these results.

In conclusion, the present study demonstrated that postoperative treatment with 0.05% CsA is effective in increasing the LLT as well as TBUT after cataract surgery. A higher preoperative LLT and 0.05% CsA treatment following cataract surgery could be significant determinants of higher postoperative LLT.

## Supporting information

S1 Checklist (DOC)

S1 Table Preoperative clinical characteristics and demographics of 50 subjects, including all subjects that were lost to follow up within 1-month after cataract surgery. (DOCX)

S1 File (DOCX)

S2 File (DOCX)
