# Supplementary material for: Efficacy of 0.05% cyclosporine A on the lipid layer and meibomian glands after cataract surgery: A randomized, double-masked study
Source: PLoS One. 2021 Jan 11;16(1):e0245329. doi: 10.1371/journal.pone.0245329 (PMC7799832; doi:10.1371/journal.pone.0245329)
Supplement: S1 Table — (DOCX) [file pone.0245329.s002.docx]

**S1 Table.** **Preoperative clinical characteristics and demographics of 50 subjects, including all subjects that were lost to follow up within 1-month after cataract surgery.**

|  | Group A | | Group B | |  |
| --- | --- | --- | --- | --- | --- |
|  | Mean (SD) | 95%, CI | Mean (SD) | 95%, CI | *p* value |
| Age (yr) | 62.37 (12.03) | 56.99, 68.91 | 67.88 (9.13) | 63.89, 72.61 | 0.245* |
| TBUT (sec) | 10.73 (1.09) | 10.27, 11.32 | 10.88 (1.29) | 10.47, 11.59 | 0.296* |
| OSDI score | 18.06 (6.39) | 14.08, 22.33 | 13.12 (8.99) | 8.48, 16.99 | 0.107* |
| Schirmer’s I test score (mm) | 14.59 (5.63) | 12.08, 17.32 | 15.69 (5.15) | 13.39, 18.03 | 0.131* |
| LLT (nm) | 78.23 (22.62) | 68.80, 86.59 | 75.68 (20.39) | 67.07, 84.93 | 0.703* |
| Follow-up duration (days) | 131.36 (26.61) | 120.49, 145.57 | 128.12 (18.14) | 120.74, 137.35 | 0.587* |
|  | N (%) | | N (%) | |  |
| Gender (male/female) | 12 (48.0) / 13 (52.0) | | 15 (60.0) / 10 (40.0) | | 0.286^#^ |
| Laterality (OD/OS) | 15 (60.0) / 10 (40.0) | | 10 (40.0) / 15 (60.0) | | 0.091^#^ |
| Upper meiboscore (grade 0/1/2/3) | 7 (28.0) / 7 (28.0) / 7 (28.0) / 4 (16.0) | | 7 (30.0) / 6 (25.0) / 8 (40.0) / 3 (5.0) | | 0.731^#^ |
| Lower meiboscore  (grade 0/1/2/3) | 7 (28.0) / 7 (28.0) / 7 (28.0) / 4 (16.0) | | 6 (24.0) / 8 (32.0) / 7 (28.0) / 3 (12.0) | | 0.823^#^ |
