# Supplementary material for: Efficacy of 0.05% cyclosporine A on the lipid layer and meibomian glands after cataract surgery: A randomized, double-masked study
Source: PLoS One. 2021 Jan 11;16(1):e0245329. doi: 10.1371/journal.pone.0245329 (PMC7799832; doi:10.1371/journal.pone.0245329)
Supplement: S1 File — (DOCX) [file pone.0245329.s003.docx]

| 연구제목 | 한국인에서 백내장 수술 후 사이클로스포린 0.05% 점안액(레스타시스^®^ SD)의 눈물 지질층 및 메이봄선 개선 효과 평가를 위한 이중 맹검, 전향적 비교 연구  A single center, double blind, prospective, comparative study on the efficacy 0.05% cyclosporine A on the lipid layer and meibomian gland after cataract surgery for the Korean patients |
| --- | --- |
| 디자인 | Single center, double blind, Prospective, Comparative Study |
| 연구대상 | 건성안 소견이 없는 백내장 수술 예정 환자 |
| 증례수 | 총 50 cases |
| 연구목적 | 본 연구는 단일기관 연구자, 피험자 이중 맹검의 전향적 연구로 설계하여 실제 임상현장에서 전향적인 데이터를 수집/분석하여 사이클로스포린 0.05% 점안액의 백내장 수술 시행 후 눈물 지질층 및 메이봄선에 미치는 임상효과를 카르복시메틸셀룰로오스나트륨 점안액과 비교를 통해 확인하고자 한다. |
| 연구의약품 | - 사이클로스포린 점안액(0.05%) – 레스타시스^®^ SD - 카르복시메틸셀룰로오스나트륨 점안액(0.5%) – 리프레쉬플러스^®^ SD |
| 선정기준 | 1. 만 19세 이상의 성인 남녀 2. Tear breakup time (TBUT) 10초 이상인 자 3. Schirmer’s type I test 10mm 이상인 자 4. 연구 참여 3개월 이내에 Cyclosporine 점안제 또는 diquafosol 계열의 인공누액, 스테로이드 점안제, 항생 점안제를 사용하지 않은 자 5. 세극등검사에서, 정상 눈꺼풀 깜빡임을 보이는 자 6. 본 연구에 참여하기로 자발적으로 동의한 자 |
| 제외기준 | 1. 쇼그렌 증후군 환자 2. 심한 안검염 환자 3. 안구 수술, 레이저 치료를 받은 자 4. 중증의 안구 염증/감염이 있는 환자 5. 건성안 치료 목적 이외에 안약을 점안 중인 환자(녹내장, 알레르기 등) 6. 연구의약품에 과민반응 환자 7. 기타 시험자가 부적합 하다고 판단한 자 |
| 병용금지약물 | 시험약과 대조약 이외의 인공누액과 다른 국소 안약, 연고 등 건성안 치료를 목적으로 하는 약제 |
| 관찰군 | - 시험군: 사이클로스포린 점안액(0.05%) – 레스타시스^®^ SD - 대조군: 카르복시메틸셀룰로오스나트륨 점안액(0.5%) – 리프레쉬플러스^®^ SD |
| 관찰 기간 | 3개월 이상 |
| 임상시험방법 | 1. 연구자 – 피험자 이중 맹검(double blind)의 전향적 연구로 진행 2. 연구자는 건성안 소견이 없는 백내장 수술 예정 환자에 대해 자발적 동의 취득 3. 방문주기   수술 전 Baseline (Visit 1) 🡪 수술 후 1개월 (Visit 2) 🡪 수술 후 3개월 (Visit 3) 🡪 수술 후 최종방문 (Visit 4) |
| 유효성 및 안전성 평가변수 | 1. 1차 유효성 평가 변수  - 수술 전 Baseline 대비 수술 후 최종방문 시점에서 Tear Breakup Time(TBUT), Schirmer’s I, Lipid layer thickness (LLT), Meiboscore, ocular surface disease index (OSDI) score 변화의 군간 비교   ***Tear Breakup Time(TBUT)*** 측정: 0.4M 플루오레신 검사지(Haag-Streit, Switzerland)를 결막낭에 점적하고 수초간 몇 번 동안 눈을 깜박이게 한 후 cobalt blue light 하에서 염색된 눈물막층에서 최초로 건조점(dry spot)이 나타나기까지의 시간을 측정한다. 3번 측정 후 평균값을 구하도록 한다.  ***Schirmer’s I*** 측정: 점안마취제 (Alcaine^®^, Alcon, Ft Worth, TX, USA) 를 점안 뒤, 5분 후에 하안검의 이측 1/3 지점에 paper strip을 위치한 후 5분 뒤에 눈물액으로 적셔진 부분의 길이를 측정하였다.  ***Lipid layer thickness (LLT)*** 및 ***Meiboscore*** 측정: LipiView^®^ Ocular Surface Interferometer(TearScience^®^ Inc, Morrisville, NC, USA)는 눈물막 지질층에서 반사되는 빛의 간섭 현상을 이용한 검사로, 나노미터 단위의 눈물 지질층 두께를 측정하고, 마이봄샘 구조를 이미지로 구현한다. 검사 전 안연고, 지질 성분이 함유 된 점안액 사용을 금지했으며, 검사 전 눈꺼풀이 영향받지 않도록 30분간 안정을 취한 후 검사를 시행하였다. 관찰기 간 동안 평균 눈물 지질층 두께를 측정했으며, LipiView^®^ 에서 얻은 마이봄샙 촬영을 분석하여, 상하안검의 마이봄샘 위축 정도에 따라 위축이 없으면 0점, 1/3 이하이면 1점, 1/3 – 2/3 이면 2점, 2/3 보다 많으면 3점으로 측정하였다.  ***ocular surface disease index (OSDI) score*** 측정: 안구건조증 증상 관련 질문 3가지, 시야 관련 질문 6가지, 환경 자극 관련 질문 3가지로 구성되어 있으며, 각 질문마다 0에서 4점으로 응답하여 각 점수의 총합을 답변한 질문 수로 나누어 전체 점수를 측정하여, 0점에서 100점으로 평가하며 점수가 클수록 증상 이 심함을 나타낸다.   1. 2차 유효성 평가 변수  - 수술 전 Baseline 대비 수술 후 1개월, 3개월, 최종 방문 시점에서 **Tear Breakup Time(TBUT), Schirmer’s I** 측정값 변화의 군별 비교분석   1차 유효성 평가 변수와 측정방법은 동일, 시간에 따른 군간 비교 목적   - 최종 방문 시점에서, lipid layer thickeness (LLT) 측정값에 영향을 미치는 수술 전 Baseline 인자 각 군별 분석   1차 유효성 평가 변수와 측정방법은 동일.   - 술 전 baseline 대비 최종 방문 시점 lipid layer thickness (LLT) 차이값에 영향을 미치는 수술 전 Baseline 인자 각 군별 분석   1차 유효성 평가 변수와 측정방법은 동일.   1. 안전성 평가 변수  - 이상반응 |
| 연구 Flow | \| Visit \| Baseline \| 1개월  ± 1주 \| 9개월  ± 1주 \| 최종  방문 \| \| --- \| --- \| --- \| --- \| --- \| \| 대상자 동의 \| O \|  \|  \|  \| \| 대상자 기초정보 \| O \|  \|  \|  \| \| 병력/수술력 \| O \|  \|  \|  \| \| 선행약물 확인 \| O \|  \|  \|  \| \| 선정/제외기준 \| O \|  \|  \|  \| \| TBUT \| O \| O \| O \| O \| \| Schirmer’s I \| O \| O \| O \| O \| \| LLT \| O \|  \|  \| O \| \| Meiboscore \| O \|  \|  \| O \| \| OSDI score \| O \|  \|  \| O \| \| 이상반응 \|  \| O \| O \| O \| |
| 유효성 및 안전성 평가변수의 통계분석 방법 | 1. 공변량 보정 및 정규분포 검정  1, 2차 유효성 평가변수 분석 시 인구통계학적 변수 및 임상병력 변수가 두 군간에 평형을 이루지 않는 경우, 해당 변수를 공변수로 포함하는 공분산분석(ANCOVA)을 실시하며, Kolmogorov–Smirnov 분석을 이용하여 유효성 평가변수에 대한 정규분포 여부를 확인한다.  2. 1차 유효성 평가변수   - 수술 전 Baseline 대비 수술 후 최종방문 시점에서 **Tear Breakup Time(TBUT), Schirmer’s I, Lipid layer thickness (LLT), Meiboscore, ocular surface disease index (OSDI) score** 변화의 군간 비교   Baseline 대비 수술 후 최종방문 시점에서 TBUT, Schirmet’s I, LLT, meiboscore, OSDI score 변화의 각 군별 비교는 Paired T-test를 이용하며, 최종방문 시점에서, 평가변수의 군간 비교는 Two-sample independent T-test를 이용하여 검정한다.  3. 2차 유효성 평가변수   - 수술 전 Baseline 대비 수술 후 1개월, 3개월, 최종 방문 시점에서 **TBUT** 측정값 변화의 군별 비교분석   각 군에서, Baseline, 수술 후 1개월, 3개월, 최종방문 시점에서 TBUT 의 시점간 비교는 반복측정 분산분석(Repeated Measure ANOVA)을 이용하여 검정한다..   - 수술 전 Baseline 대비 수술 후 1개월, 3개월, 최종 방문 시점에서 **Schirmer’s I** 측정값 변화의 군별 비교분석   각 군에서, Baseline, 수술 후 1개월, 3개월, 최종방문 시점에서 Schirmer’s I 의 시점간 비교는 반복측정 분산분석(Repeated Measure ANOVA)을 이용하여 검정한다..   - 최종 방문 시점에서, lipid layer thickeness (LLT) 측정값에 영향을 미치는 수술 전 Baseline 인자 각 군별 분석   각 군에서, 수술 후 최종 방문 LLT 측정값에 영향을 미치는 생체 인자 및 수술 전 인자에 대한 분석은 다중회귀분석을 통해 검정한다.   - 술 전 baseline 대비 최종 방문 시점 lipid layer thickness (LLT) 차이값에 영향을 미치는 수술 전 Baseline 인자 각 군별 분석   각 군에서, 수술 후 최종 방문 LLT 측정값에 영향을 미치는 생체 인자 및 수술 전 인자에 대한 분석은 다중회귀분석을 통해 검정한다.   1. 안전성 평가 변수  - 이상반응   이상반응 발현 임상시험대상자 수, 발현율 및 발현건수 등을 제시하고, 군간 차이를 Pearson`s Chi-square test 또는 Fisher`s Exact test를 이용하여 검정한다. |
| 시험 기간 | IRB 승인일로부터 12개월 |
