# Supplementary material for: Efficacy of 0.05% cyclosporine A on the lipid layer and meibomian glands after cataract surgery: A randomized, double-masked study
Source: PLoS One. 2021 Jan 11;16(1):e0245329. doi: 10.1371/journal.pone.0245329 (PMC7799832; doi:10.1371/journal.pone.0245329)
Supplement: S2 File — (DOCX) [file pone.0245329.s004.docx]

| Title | A single-center, double-masked, prospective, comparative study on the efficacy of 0.05% cyclosporine A on the lipid layer and meibomian gland after cataract surgery on Korean patients |
| --- | --- |
| Study design | Single-center, double-masked, prospective, comparative study |
| Participants | Patients scheduled for cataract surgery with no evidence of dry eyes. |
| Subjects | A total of 50 subjects. |
| Objective | This is a single-center, double-masked, prospective, and comparative study that aims to determine the effects of 0.05% cyclosporine eye drops on tear film lipid layers and meibomian glands after cataract surgery, compared to those of carboxymethyl cellulose eye drops, by collecting and analyzing data obtained during clinical practice. |
| Study drug | - Cyclosporine eye drops (0.05%): RESTASIS ^®^SD - Carboxymethyl cellulose sodium eye drops (0.5%): REFRESH PLUS ^®^SD |
| Criteria for inclusion | In order to be eligible for participation in this study, the subject had to:   1. Be male or female, aged 19 years or older; 2. Have a tear breakup time (TBUT) of 10 seconds or more; 3. Have a Schirmer’s I test result of 10 mm or more; 4. Have not used cyclosporine eye drops, diquafosol-based artificial drops, steroid eye drops, or antibiotic eye drops within 3 months before participation; 5. Show normal blinking during a slit lamp exam; 6. Voluntarily agree to participate in this study. |
| Criteria for exclusion | The subject was excluded from the study if the subject either:   1. Has Sjögren's syndrome; 2. Has severe blepharitis; 3. Had ocular surgery or laser eye surgery; 4. Has severe ocular inflammation/infection; 5. Is using eye drop treatment for dry eyes, such as glaucoma or allergies; 6. Shows sensitivity to study drugs; 7. Is considered to be ineligible for participation owing to reasons other than the aforementioned exclusion criteria based on the judgment of the principal researcher. |
| Prohibited concomitant medications | Artificial tear drops or topical medications for the treatment of dry eyes other than the study drugs. |
| Observation group | - Experimental group: Cyclosporine eye drops (0.05%): RESTASIS ^®^SD - Control group: Carboxymethyl cellulose sodium eye drops (0.5%): REFRESH PLUS^®^SD |
| Observation period | For more than 3 months |
| Study methods | 1. Double-masked, prospective study; 2. Obtain voluntary consent from patients who are scheduled to undergo cataract surgery and without evidence of dry eyes; 3. Visit periods   Preoperative visit: assess baseline characteristics (first visit);  🡪 1 month after surgery (second visit);  🡪 3 months after surgery (third visit);  🡪 Final visit (final, fourth visit). |

| Assessment variables for efficacy and safety | 1. Primary efficacy variables  - Comparisons of **Tear Breakup Time (TBUT), Schirmer’s I test score, Lipid Layer Thickness (LLT), Meiboscore, and ocular surface disease index (OSDI) score** changes between the preoperative and last postoperative visit between the control and experimental group   ***Tear Breakup Time (TBUT) measurements***  Add 0.4 M fluorescein paper (Haag-Streit, Switzerland) to the conjunctival sac and allow the patient to blink several times. Measure the time until the appearance of the first dry spot in the dyed tear film under cobalt blue light. Calculate the average value after measuring three times.  ***Schirmer’s I test measurements***  Add anesthetic eye drops (Alcaine^®^, Alcon, Ft Worth, TX, USA) 5 minutes before the placement of a paper strip on the lateral 1:2 point of the lower eyelid. Measure the length of the wet part of the paper strip after 5 minutes of placement.  ***Lipid layer thickness (LLT) and meiboscore measurements***  A LipiView^®^ ocular surface interferometer (TearScience^®^ Inc., Morrisville, NC, USA) measures the thickness of the lipid layer in nanometers and visualizes the meibomian gland structure by assessing optical interference patterns produced by reflected light from the tear film lipid layer. The use of eye drops containing lipid substances was prohibited before testing. The patient was stabilized for 30 minutes before testing to minimize effects due to eyelids. During the observation period, we measured the average thickness of the tear film lipid layer and analyzed the image of the meibomian glands obtained by LipiView^®^. Grades were assigned according to the degree of atrophy of the meibomian glands in the upper and lower eyelids. Zero points were assigned if there was no atrophy, 1 point if atrophy was less than one third, 2 points if between one and two thirds, 3 points if over two thirds.  ***Ocular surface disease index (OSDI) score measurements***  The OSDI score assesses a total of 12 items related to dry eyes. Subjects rate their responses on a scale ranging from 0 to 4 with 0 corresponding to “none of the time” and 4 corresponding to “all of the time.” A final score is calculated ranging from 0 to 100, with scores 0 to 12 representing normal, 13 to 22 representing mild dry eye disease, 23 to 32 representing moderate dry eye disease, and greater than 33 representing severe dry eye disease.   1. **Secondary efficacy variables**  - Comparison of changes in **TBUT and Schirmer’s I test score** during the follow-up period between the control and experimental groups   Efficacy variables and the corresponding method of assessment are identical to the primary efficacy variables.   - At the final visit, an analysis of baseline factors that affect lipid layer thickness (LLT) was made in each group.   Efficacy variables and the corresponding method of assessment are identical to the primary efficacy variables.   - Analysis of baseline factors affecting the amount of changes in lipid layer thickness (LLT) from baseline to the last visit in each group was made.   Efficacy variables and the corresponding method of assessment are identical to the primary efficacy variables.   1. Safety variables  - Adverse drug reactions. |
| --- | --- |
| Study Flow | \| Visit \| Baseline \| 1 month  ±1 week \| 3 months  ± 1 week \| Final visit \| \| --- \| --- \| --- \| --- \| --- \| \| Subject consent \| O \|  \|  \|  \| \| Basic information of subject \| O \|  \|  \|  \| \| Medical/Surgical history \| O \|  \|  \|  \| \| Concomitant medications \| O \|  \|  \|  \| \| Inclusion/exclusion criteria \| O \|  \|  \|  \| \| TBUT \| O \| O \| O \| O \| \| Schirmer’s I test score \| O \| O \| O \| O \| \| LLT \| O \|  \|  \| O \| \| Meiboscore \| O \|  \|  \| O \| \| OSDI score \| O \|  \|  \| O \| \| Adverse drug reactions \|  \| O \| O \| O \| |
| Statistical analysis of efficacy and safety assessment variables | 1. Adjusted covariance and normality verification  When demographic variance is not equal between groups, ANCOVA is used to integrate unmatched variants to covariants, and a normality test is performed via Kolmogorov-Smirnov analysis.  2. Primary efficacy variables   - Comparisons of **Tear Breakup Time (TBUT), Schirmer’s I test score, lipid layer thickness (LLT), meiboscore, and ocular surface disease index (OSDI) score** changes from the preoperative to the last postoperative visit between the control and experimental group.   At the final visit, the analysis of covariance (ANCOVA) is used to compare efficacy variables (**TBUT, Schirmer’s I test score, LLT, meiboscore, and OSDI score**) between the groups.  3. Secondary efficacy variables   - Comparisons of **TBUT and Schirmer’s I test scores** during the follow-up period between the control and experimental groups.   Repeated measures ANOVA is used to compare TBUT and Schirmer’s I test scores in each group upon each preplanned visit (preoperative, second, third, and last visits).   - In the final visit, an analysis of baseline factors that affect lipid layer thickness (LLT) was made in the study.   Multiple regression analysis is done.   - Analysis of baseline factors affecting the amount of changes in lipid layer thickness (LLT) from baseline to the last visit is made in each group.   Multiple regression analysis is done.   1. Safety variables  - Adverse drug reactions.   Results related to adverse reactions are presented, including the number of subjects with adverse reactions, incidence rates of adverse reactions, and cases of adverse reactions. Pearson’s chi-square test or Fisher’s exact test is used to analyze differences between groups. |
| Experimental period | 12 months from IRB approval |
